# P-1527. Growth rate comparisons for clinical Escherichia coli (EC) isolates in various media stratified by inner colony (IC) production during fosfomycin (FOF) disk diffusion (DD) testing

**DOI:** 10.1093/ofid/ofaf695.1708

**Published:** 2026-01-11

**Authors:** Tiffany Chang, Lindsey Collins, Jenna Salay, Khadijah Malik, Alexis J Walters, Aalunya A Teso, Katlin K Her, Morgan L Bixby, Elizabeth B Hirsch

**Affiliations:** University of Minnesota, Twin Cities, Minneapolis, MN; University of Minnesota College of Pharmacy, Minneapolis, Minnesota; University of Minnesota College of Pharmacy, Minneapolis, Minnesota; University of Minnesota College of Pharmacy, Minneapolis, Minnesota; University of Minnesota College of Pharmacy, Minneapolis, Minnesota; University of Minnesota College of Pharmacy, Minneapolis, Minnesota; University of Minnesota College of Pharmacy, Minneapolis, Minnesota; Univeristy of Minnesota, Saint Paul, Minnesota; University of Minnesota College of Pharmacy, Minneapolis, Minnesota

## Abstract

**Background:**

Recent studies have highlighted the frequent presence of IC during FOF DD testing. It is assumed that increased resistance comes with fitness trade-off; however, our previous work in *Klebsiella pneumoniae* suggests *in vitro* fitness of IC are highly similar to IC-producers (IC-P) from which they arose. Therefore, we compared growth rates of susceptible EC never-producers (NP), IC-P, and their non-susceptible IC alone and together in various media to compare *in vitro* fitness differences.

**Figure:**
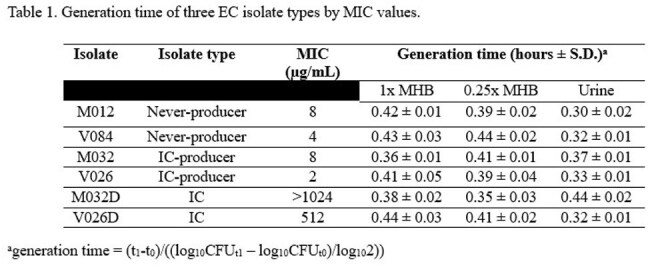

**Figure:**
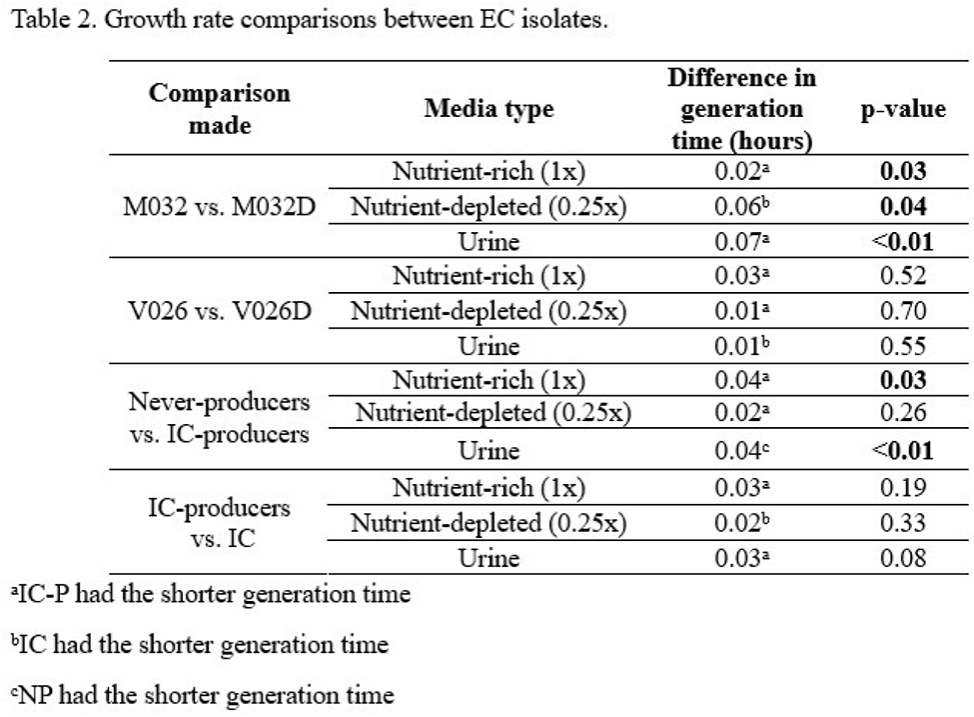

**Methods:**

Single-isolate time growth (sTG) and competition TG (cTG) were conducted over 24 hours and 72 hours, respectively. An initial inoculum of 10⁵ CFU/mL was used for sTG in nutrient-rich Mueller-Hinton broth (1× MHB), nutrient-depleted (0.25× MHB), and filter-sterilized pooled human urine where CFU/mL was serially measured. For cTG, inoculum of equal parts IC-P and IC was added to a flask of 1x MHB at t_0_, with media replaced every 12 hours. CFU measurements were taken in technical duplicate, and experiments were run in biological duplicate on separate days. In cTG, selective plating (FOF+/FOF-) was used to determine the burden of IC-P and IC separately. Generation times during logarithmic growth were calculated. T-test comparisons were performed between individual IC-P and IC pairs, IC-P and IC as a set, and NP and IC-P as a set.

**Figure:**
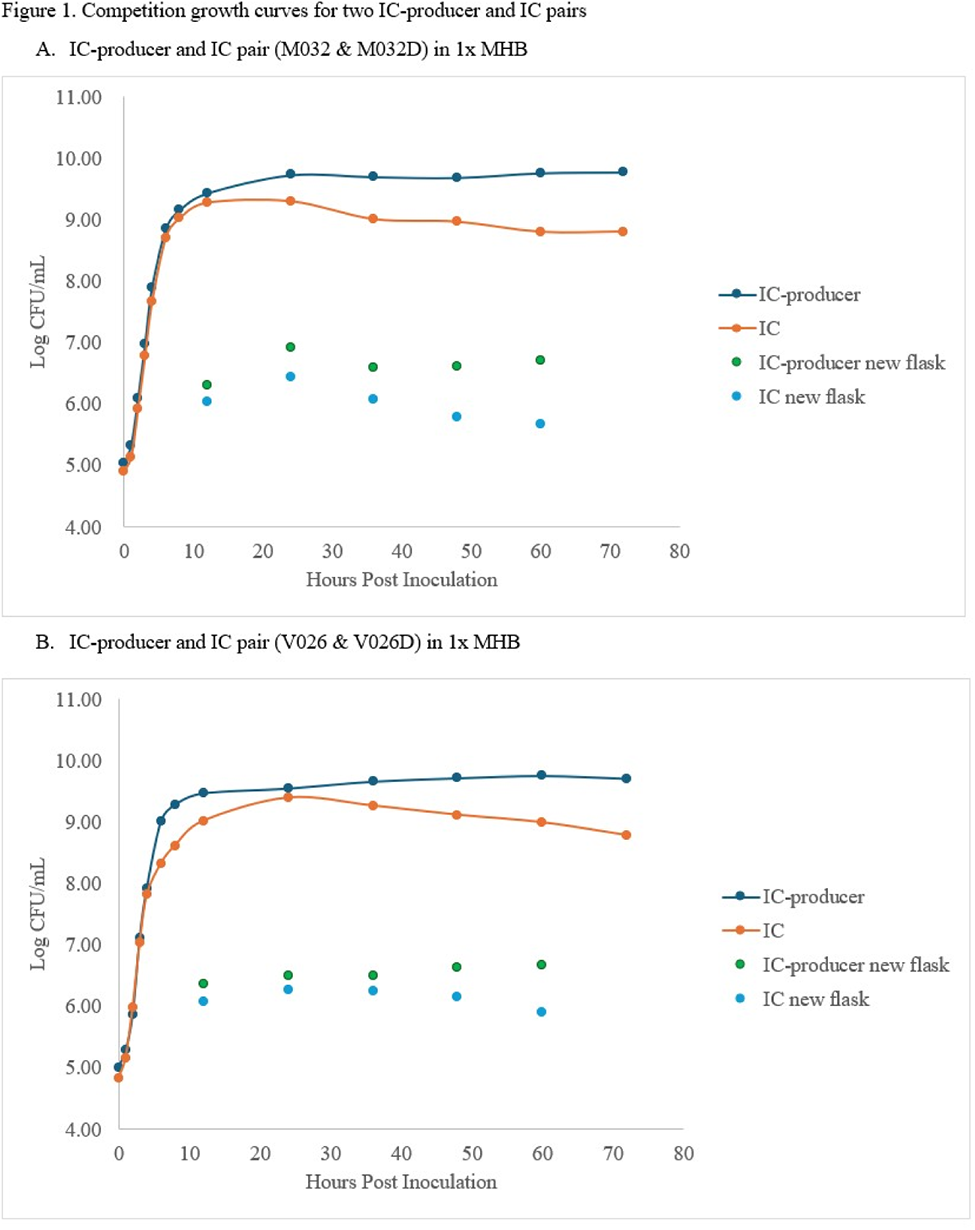

**Results:**

Generation times ranged from 0.36 to 0.44, 0.35 to 0.44, and 0.30 to 0.44 hours in 1x MHB, 0.25x MHB, and urine, respectively, and there was no consistent pattern for which isolates exhibited longer generation times (Table 1). There were no significant differences in generation times except for the M032/M032D isolate pair for all media conditions and between NP and IC-P in 1x MHB and urine conditions (Table 2). For isolate pairs M032/M032D and V026/V026D, the difference in log CFU/mL was 0.16 and 0.45 at hour 12 and remained < 1 through hour 72 (Figure 1).

**Conclusion:**

There were no significant differences in generation time between IC-P and IC as a set for sTG and the cTG curves were within 1 log CFU/mL from hour 12 and beyond, signifying no significant *in vitro* fitness loss in IC, regardless of media condition. This study justifies further investigations into resistance mutations for isolate pairs via whole genome sequencing and validation in a murine urinary tract infection model.

**Disclosures:**

Tiffany Chang, MS, Gilead Sciences: Stocks/Bonds (Public Company) Elizabeth B. Hirsch, PharmD, FCCP, FIDSA, GSK: Advisor/Consultant|GSK: Honoraria

